# Transcriptome analysis of anti-fatty liver action by Campari tomato using a zebrafish diet-induced obesity model

**DOI:** 10.1186/1743-7075-8-88

**Published:** 2011-12-13

**Authors:** Toshiyuki Tainaka, Yasuhito Shimada, Junya Kuroyanagi, Liqing Zang, Takehiko Oka, Yuhei Nishimura, Norihiro Nishimura, Toshio Tanaka

**Affiliations:** 1 Department of Molecular and Cellular Pharmacology, Pharmacogenomics and Pharmacoinformatics, Mie University Graduate School of Medicine, Mie, Japan; 2 Mie University Medical Zebrafish Research Center, Mie, Japan; 3 Department of Bioinformatics, Mie University Life Science Research Center, Mie, Japan; 4 Department of Omics Medicine, Mie University Industrial Technology Innovation Institute, Mie, Japan; 5 Department of Translational Medical Science, Mie University Graduate School of Medicine, Mie, Japan; 6 Delica Foods Co Ltd, Tokyo, Japan

**Keywords:** dyslipidemia, liver steatosis, vegetables, diet-induced obesity, zebrafish, DNA microarray

## Abstract

**Background:**

High dietary intake of vegetable products is beneficial against obesity and its related diseases such as dyslipidemia, nonalcoholic fatty liver disease, and cancer. We previously developed a diet-induced obesity model of zebrafish (DIO-zebrafish) that develops visceral adiposity, dyslipidemia, and liver steatosis. Zebrafish is a polyphagous animal; thus we hypothesized that DIO-zebrafish could be used for transcriptome analysis of anti-obesity effects of vegetables.

**Results:**

Each vegetable exhibited different effects against obesity. We focused on "Campari" tomato, which suppressed increase of body weight, plasma TG, and lipid droplets in livers of DIO-zebrafish. Campari tomato decreased *srebf1 *mRNA by increase of *foxo1 *gene expression, which may depend on high contents of β-carotene in this strain.

**Conclusions:**

Campari tomato ameliorates diet-induced obesity, especially dyslipidemia and liver steatosis via downregulation of gene expression related to lipogenesis. DIO-zebrafish can discriminate the anti-obesity effects of different strains of vegetables, and will become a powerful tool to assess outcomes and find novel mechanisms of anti-obesity effects of natural products.

## Background

Dramatic increases in the occurrence of obesity are a severe problem in developed countries. The World Health Organization estimates that 310 million people worldwide are obese [1]. Obesity is associated with several adverse health consequences including type 2 diabetes mellitus, dyslipidemias, nonalcoholic fatty liver and gallstones, cardiovascular disease (CVD), Alzheimer's disease, and certain types of cancer [2]. Recently, growing evidence from several epidemiological and clinical studies has indicated health benefits of certain kinds of vegetables against obesity and its related diseases. For example, tomato and its components could lower plasma cholesterol and triacylglyceride (TG) [3,4] and may prevent obesity-related diseases including atherosclerosis and CVD [5-7], hypertension [8], and nonalcoholic steatohepatitis-promoted liver cancer [9]. Since the components of vegetables greatly vary depending on the strain, production area, and agricultural method, it is very difficult to evaluate how these parameters affect the clinical condition of obesity. To evaluate the anti-obesity effects of different vegetable components rodent models of obesity such as ob/ob mouse have been used *in vivo*. Although rodent models have greatly contributed to our understanding of human obesity [10], experiments using rodent models require considerable time and infrastructural support and are relatively expensive. Zebrafish, a small teleost, offers a powerful vertebrate model for human diseases. The high degree of genetic conservation in comparison with mammals contributes to its emergence as a model for obtaining insights into fundamental human physiology.

We constructed diet-induced obesity model of zebrafish (DIO-zebrafish) overfed with *Artemia *as high-fat diet [11]. Increases of body weight, plasma TG, and liver steatosis seen in this model are highly consistent with obesity observed in humans and rodent models of DIO. The histological configuration of target organs of adiposity such as liver and visceral fat is also similar [11,12]. Furthermore, main gene expression profile of visceral fat is also in common with human [11]. There are several advantages in the DIO-zebrafish model. The response of zebrafish to *Artemia *is very good; almost all zebrafish overfed with these organisms become obese, with more homogeneity than rodent models. DIO-zebrafish is easy to create and takes only 2 weeks to induce obesity. In addition, zebrafish is a polyphagous animal allowing *in vivo *screening through oral administration of test compounds. In our preliminary study zebrafish could ingest many kinds of vegetables including pumpkin, eggplant, cucumber, green pepper, and broccoli, grains including rice, and beans as a mixture of ordinary fish food, for example, Tetramin^®^. Thus zebrafish can become a suitable animal model in feeding experiment to evaluate influences of food compositions against human obesity.

## Methods

### Ethical approval

The investigation conformed to the ethical guidelines established by the Institutional Animal Care and Use Committee of Mie University.

### Materials

Tomatoes (Delica strain), pumpkins (Ebisu and Kurimasaru strains), and egg plants (Choshi and Senryo) were purchased from Delica Foods (Tokyo, Japan). Since the Delica strain is a red-type tomato that is widely available in Japanese supermarkets, we defined it as the "regular" tomato. Campari tomatoes were purchased from IDE Farm (also called Shio-Tomato, Kumamoto, Japan). Pictures of these vegetables are shown in Additional file 1, Figure S1. Lycopene (L9879) was purchased from Sigma-Aldrich (St. Louis, MO, USA)

### Preparation of vegetable and lycopene-containing fish foods

First, each vegetable was homogenized to a liquid phase using a standard blender intended for at-home use (MX-X37, National, Japan). The vegetable juice was stored overnight at -80°C. The frozen juice was then lyophilized (DC-400, YAMATO SCIENTIFIC, Japan). When drying was complete, the same volume (weight) of water and commercial flake food (Tetramin tropical flakes, Tetra, Germany) were added to the vegetable powder and blended together, resulting in a mixture containing 50% freeze-dried vegetable powder. After that, the mixture was stored at -80°C and then lyophilized again. To make the lycopene-containing fish food, lycopene was suspended in ethanol and mixed with Tetramin to a final concentration of 0.74 μg/ml. After drying, it was carefully ground to granules (not to powder) using a mortar and pestle. To adjust the granule size to be suitable for consumption by adult zebrafish, the grinding process was repeated until all of the granules could pass through a 700 μm mesh sieve. Granules were purged with nitrogen gas, protected from light, and stored in aliquots at 4°C prior to use. Tetramin, homogenized once with water and then lyophilized was used as the control fish food.

### Feeding zebrafish and experimental design

**Figure 1:**
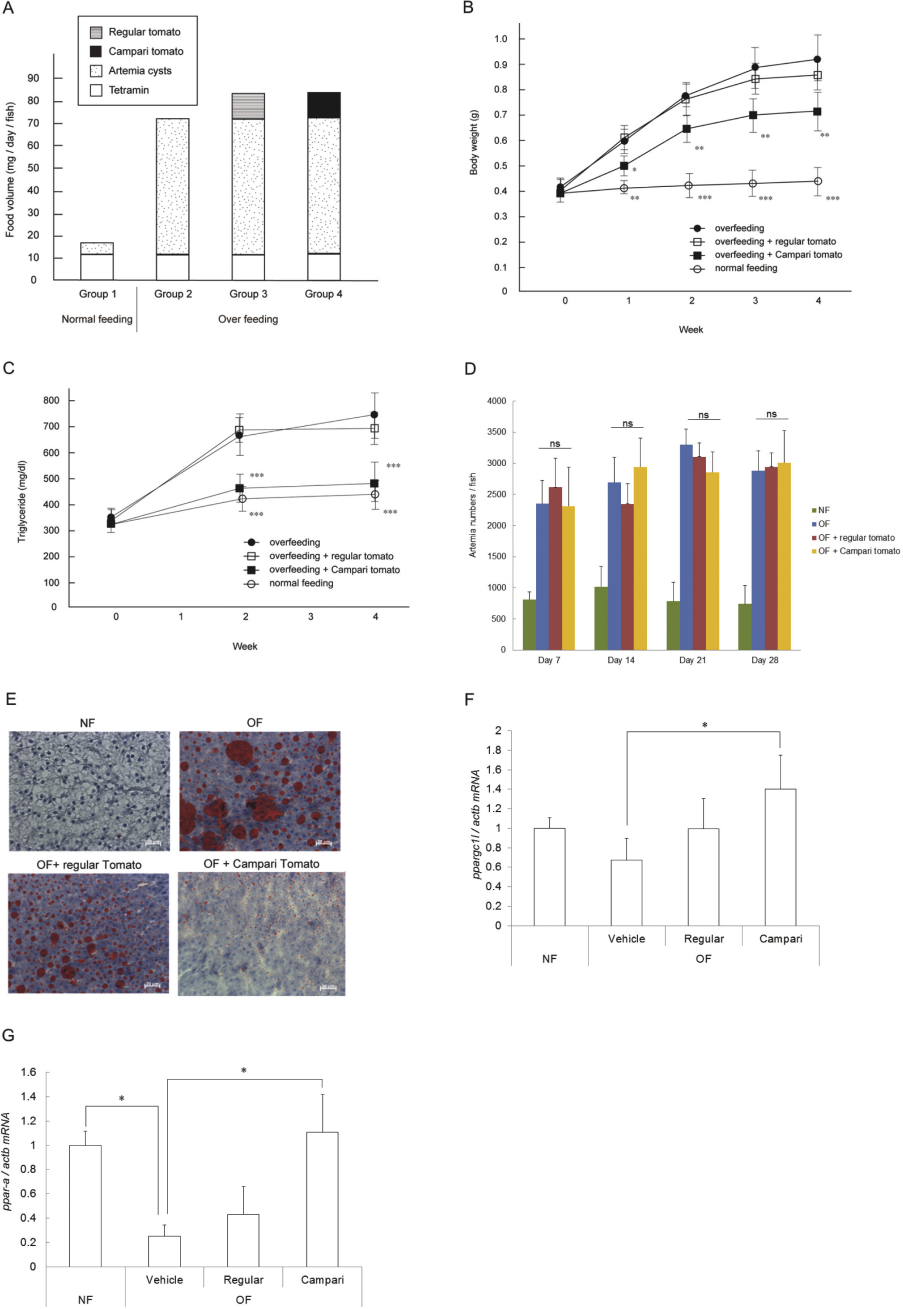
**Assessment of body weight, plasma TG, and hepatic steatosis in zebrafish overfed with Campari and regular tomatoes**. (A) Feeding condition of each group. Group 1, normal feeding; group 2, overfeeding of *Artemia *with Tetramin (vehicle); group 3, overfeeding with regular tomato; group 4, overfeeding with Campari tomato. Feeding experiments were conducted over 2 and 4 weeks. (B) Average body weight in each group during 4-week feeding experiments. Values are mean ± SD. Each group contained 20 samples. **P < 0.05; ***P < 0.01 vs. vehicle with overfeeding, black circles. (C) Change of plasma TG levels in the each group. ***P < 0.01 vs. vehicle with overfeeding, black circles. (D) Feeding behaviour assay counting *Artemia *numbers 90 min after feeding at 2 weeks. No significant difference was observed among vehicle, regular tomato, and Campari tomato administration in overfeeding groups. (E) Oil red O staining of liver sections. Campari tomato reduced the number and size of lipid droplets (red) compared with overfeeding and overfeeding regular tomato. (F and G) qPCR-assessed gene expression in the livers of DIO and normally fed zebrafish. Expression of *ppar-ab *(F) and *ppargc1-like *(G), a zebrafish homolog of human *PPAR-α *and *PPARGC1 *(*PGC-1α*, was normalized to actb expression. Values are mean ± SE (n = 5/group). *P < 0.05 vs. vehicle with overfeeding (OF).

Adult zebrafish (*AB*, ZIRC, Eugene, OR, USA) were kept at 28°C under a 14-h light:10-h dark cycle, and water conditions of environmental quality were maintained according to the *Zebrafish Book *[13] . Zebrafish were assigned into each dietary group for 2 or 4 weeks with 5 fish/1.7-L tank. Zebrafish in the overfeeding group were fed three times/day with *Artemia *(60 mg cysts/fish/day; Miyako Kagaku, Tokyo, Japan). Zebrafish in the control group were fed once daily in the morning with *Artemia *(5 mg cysts/fish/day) from 3.5 months postfertilization (mpf). Zebrafish were fed vegetable-containing flake food (2 mg/day) 3 times at 20 min before *Artemia *feeding during experiments (Figure 1A).

### Measurement of body weight, plasma TG and blood glucose

Body weight and length of zebrafish were measured weekly throughout the study. Length of zebrafish was measured from tip to the end of the body. For the blood chemistry analyses, zebrafish were deprived of food overnight and blood was withdrawn from the dorsal artery by heparinized glass capillary needle (GD-1; Narishige, Tokyo, Japan) at the indicated times. Blood glucose was determined using a hand-held blood glucose meter (Glutest Neo, Sanwa Kagaku Kenkyusho, Nagoya, Japan). For determination of plasma TG, the blood samples were centrifuged for 3 min at 3, 500 rpm at room temperature and the plasma harvested; triglycerides were measured using a Wako L-type TG kit (Wako Pure Chemical Industries, Tokyo, Japan) according to the manufacturer's protocol.

### Feeding volume assay

Feeding volume of *Artemia *was measured weekly throughout the study. Hatched *Artemia *(5 or 60 mg cysts/fish/day) was fed to zebrafish in 1.7-L fish tank as described above. For blank (no fish) control, *Artemia *was put in a blank 1.7-L tank without zebrafish (only breeding water). After 2 h, remaining numbers of *Artemia *not eaten by zebrafish were counted three times and subtracted from that in control fish tank to determine feeding numbers of *Artemia *in each tank.

### Carotenoids, sugar contents, ascorbic acid, and NO_3_^- ^determination

Among anti-obesity and antidyslipidemic components of tomatoes, antioxidant substances such as lycopene [14-16] and β-carotene [17-19] are well known. Carotenoid extraction and analysis were performed as previously described [20]. Samples were homogenized by mixer for home use. For each sample, 3 g was placed in a 50-ml brown glass centrifuge tube and homogenized in 35 ml of extraction solvent including diethylether/methanol (7:3, v/v) (Wako) by homogenizer (AM-7; Nihonseiki, Tokyo, Japan). After residue settling, the extract was removed to a 100-ml brown glass flask. After adding 15 ml of extraction solvent, the residue was again homogenized. After settling, the extract was removed to the brown flask. The extraction step, consisting of 15-ml solvent additions, was repeated. Extracts were combined and the final volume was adjusted to 100 ml. Extracted samples were filtered by 0.2-μm GL Chromatodisk 13N disposable filter (GL Science, Tokyo, Japan) then stored in vials for HPLC at -20°C before analysis. For HPLC analysis (Prominence; Shimadzu, Kyoto, Japan) isocratic separation was achieved by C18-5B column (Showa Denko, Tokyo, Japan). The mobile phase was 100% methanol for HPLC (Wako) at a flow rate of 1.0 ml/min. Sample injection volume was 10 μl and column temperature 25°C; peaks were detected at 450 nm. All analyses were performed in duplicate, and quantification of carotenoid isomers was carried out by comparing retention times to analytical standards of lycopene (Sigma-Aldrich) and β-carotene (Wako). Total sugar content expressed as brix was determined by refractometer (PAL-1; Atago, Tokyo, Japan). Ascorbic acid (vitamin C) was quantified by reflectometer (RQflex plus10; Merck, Darmstadt, Germany). Two hundred grams of samples were homogenized with metaphosphoric acid (Wako) to avoid ascorbic acid oxidation using mixer for home use. The samples were filtered by paper filter and used for measurements. NO_3_^- ^was quantified by RQflex plus10. One hundred grams of samples were homogenized with equal volume of deionized water using mixer for home use. The samples were filtered by paper filter and used for measurements.

### Measurement of the DPPH radical scavenging activity of tomato-containing fish foods

The 1, 1-Diphenyl-2-picrylhydrazyl (DPPH) radical scavenging activities of tomato-containing fish foods were measured using a previously reported method [21]. In brief, a portion of the crude material was dissolved in 200 μl EtOH, mixed with 800 μl of 100 mM Tris-HCl buffer (pH 7.4)), and then added to 1 ml of 500 μM DPPH in EtOH. The mixture was shaken vigorously and left for 20 min at room temperature in the dark. The DPPH radical scavenging activity of a 60 μl sample of the reaction mixture was determined using reversed-phase HPLC analysis. Analyses were done in a TSKgel Octyl-80TsQA column (4.6 × 250 min, Tosoh, Tokyo, Japan) at ambient temperature with a mobile phase of MeOH/H2O (7:3, v/v) at a flow rate of 0.8 ml/min. The peaks were monitored with a UV detector set at 517 nm. Percentage inhibition of the discolouration of DPPH by the sample extract was expressed as Trolox equivalents per 100 gram (μmol TE/100 g) [22].

### Oil Red O staining

Liver tissues were collected from zebrafish by surgical manipulation under a stereoscopic microscope (MZ16F; Leica Microsystems, Wetzlar, Germany). Livers were fixed using 10% buffered formalin solution (Histo-Fresh; Pharma, Tokyo, Japan). The fixed samples were placed in sucrose solution (Wako) at 4°C for 3 h then rapidly frozen in liquid nitrogen-cooled isopentane (Wako), embedded in Tissue-Tek (Sakura Finetek, Tokyo, Japan), and dissected by cryostat (Microm HM-550; Thermo Fisher Scientific, Waltham, MA). The sections were immersed in a working solution of Oil Red O (Wako) for 15 min and rinsed with distilled water as described previously [23]. Sections were also counterstained with Mayer's hematoxylin (Wako) to visualize the nuclei according to manufacturer's protocol.

### DNA microarray analysis

Liver tissues were collected from DIO-zebrafish after Campari and regular tomato feeding. Livers were fixed in RNA-later (Applied Biosystems, Foster City, CA, USA) at 4°C for 1 day. Then, the liver tissues were immersed in 1 ml of Isogen (NipponGene, Tokyo, Japan) and homogenized by Mixer Mill MM 300 (Retsch, Haan, Germany) with 5-mm zirconia beads (BioMedical Science, Tokyo, Japan) at 25 Hz for 3 min. After homogenization, total RNAs were extracted according to protocol of Isogen in combination with cleanup protocol of RNeasy Mini Kit (Qiagen, Hilden, Germany). The volumes of total RNA were quantified by spectrophotometer (NanoDrop ND-100; Wilmington, DE, USA). Three hundred nanograms of total RNA were converted into labeled cRNA by Low RNA input linear Amplification Kit PLUS Two-Color (Agilent Technologies, Santa Clara, CA, USA). Reverse transcription labeling with cyanine 3 (Cy3)- or cyanine 5 (Cy5)-dCTP and hybridization of the DNA microarray (Agilent Zebrafish Whole Genome Microarrays; G2518A) were carried out according to the manufacturer's protocol. To avoid a dye-specific hybridization preference for each RNA sample, cyanine dye-swap hybridization was performed. In detail, in the first series of experiments RNAs from fish overfed with regular tomato and Campari tomato fractions were labeled with Cy3 and Cy5, respectively, and hybridized to zebrafish DNA microarray. In the second series of experiments RNAs from fish overfed with regular tomato and Campari tomato fractions were labeled with Cy5 and Cy3, respectively, and used for hybridization. The microarrays were scanned by Agilent Microarray Scanner G2565BA and analyzed using Feature Extraction software (Agilent Technologies). The LOWESS (LOESS) method was applied to filtering and normalization of the data. Filtering was performed according to default algorithms of Feature Extraction software. Microarray experiments were carried out in triplicate to verify their reproducibility using RNAs isolated from different animals. Identification of gene sets differentially expressed in microarray analysis was carried out by one-way ANOVA.

### Quantitative RT-PCR

Total RNA of each sample was purified as described above. First-strand cDNA was prepared with 500 ng of total RNA using Super Script III First-strand System (Invitrogen, Carlsbad, CA, USA) with random primer (Invitrogen). Quantitative RT-PCR was performed with Power SYBR Green Master Mix (Applied Biosystems) in triplicate according to manufacturer's protocol. The sequences of the primers are shown in Additional file 2, Table S1. Data were normalized by the quantity of actin beta (actb, NM_131031); this allowed us to account for any variability in the initial template concentration as well as the conversion efficiency of reverse transcription reaction.

### Statistical analysis

All data are presented as mean ± SEM. Differences between 2 groups were examined for statistical significance by Student's *t*-test. For multiple comparisons, one-way ANOVA followed by Bonferroni-Dunn multiple-comparison procedure was used. A *P*-value < 0.05 was considered to denote statistical significance.

## Results and Discussion

### Campari tomato exhibits high anti-obesity effects in DIO-zebrafish

**Figure 2:**
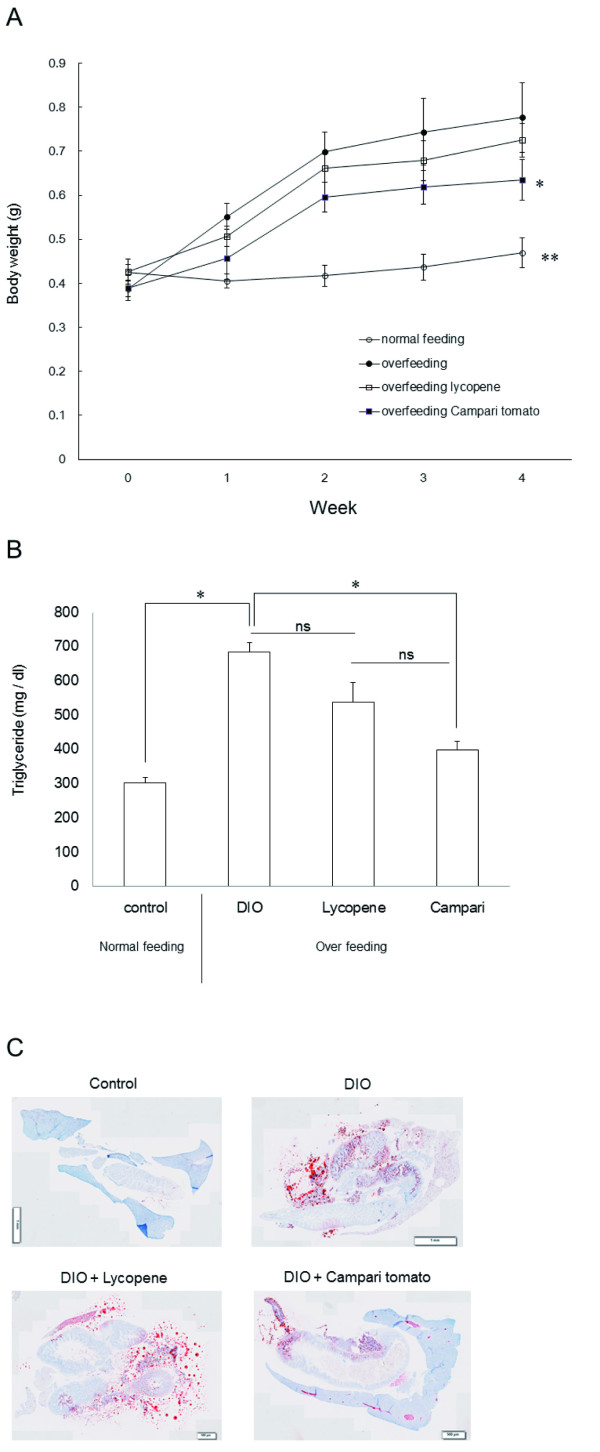
**Lycopene effects on body weight, plasma TG, and hepatic steatosis in DIO-zebrafish**. (A) Average body weight in each group during 4-week feeding experiments. Values are mean ± SD. Each group contained 20 samples. *P < 0.05; **P < 0.01 vs. vehicle with overfeeding, black circles. (B) Change of plasma TG levels in each group. *P < 0.05 vs. vehicle with overfeeding. (C) Oil red O staining of liver sections.

Compared with flake foods that have also been used to feed zebrafish [13] the amounts of fat and protein in *Artemia *are higher and lower, respectively, whereas the amount of carbohydrate is comparable [24]. Zebrafish fed 5 or 60 mg of freshly hatched *Artemia *daily consumed about 80% and 50% of the provided *Artemia*, respectively, translating to 20 and 150 cal, respectively. Since maintenance energy requirement for zebrafish is < 30 cal [25], it seems reasonable to induce DIO-zebrafish by this overfeeding protocol. After 2 weeks, increases of body weight and plasma TG were noted (Additional file 3, Table S2). In all experiments, overfed fish significantly increased body weight and plasma TG in comparison with normal feeding group (*P *< 0.01), indicating that DIO-zebrafish was well constructed. Eggplant of the "Choshi" strain showed a trend towards suppression of the diet-induced body weight increase (*P *< 0.1) and significantly reduced the increase in plasma TG (*P *< 0.05). The Choshi strain is a darker eggplant, and contains more anthocyanins than the Senryo strain (data not shown). Anthocyanins have been reported to normalize the lipid parameters in a high fat diet-induced mouse model of obesity [26], and we hypothesize that the same mechanism may be occurring in DIO-zebrafish fed Choshi eggplant-containing food. Campari strain tomato, on the other hand, dramatically suppressed increase of body weight and plasma TG in overfed fish, to almost those of normal feeding fish, in both 2- and 4-week feeding experiments (Additional file 3, Table S2, Figure 1B and 1C). However, the fasting blood glucose did not significantly differ between the Campari-fed group and the controls (Additional file 4, Figure S2). There was no appetite suppression by Campari tomato during the feeding experiment (Figure 1D). Campari tomato reduced lipid accumulation (numbers and size of red spots) of liver tissues more than overfeeding with regular tomato (Figure 1E), corresponding to the results of plasma TG lowering. In addition, since the OF fish that were fed Campari tomato-containing food ate almost the same number of calories as those in the other two OF groups, but gained less body weight, we hypothesized that their energy expenditure must be increased. In fact, genes involved in fatty acid oxidation such as peroxisome proliferator-activated receptor gamma coactivator 1α (*ppargc1a-like*, a zebrafish homolog of human PPARGC1A, also called PGC-1α) and peroxisome proliferator-activated receptor αb (*ppar-ab*), were also increased in OF Campari tomato-fed fish in comparison to OF controls (Figure 1F and 1G). Lycopene, the pigment that gives tomatoes their red colour, may play a role in preventing diseases related to obesity including dyslipidemia [14-16]. Campari tomatoes contain more lycopene than regular tomatoes (Table 1). This results in their high antioxidant activity, as measured by their DPPH radical scavenging activity (Additional file 5, Figure S3). In a preliminary experiment 4-week administration of lycopene at a volume based on the amount in Campari tomatoes (0.74 μg/mg diet) slightly and nonsignificantly decreased weight gain (Figure 2A) and plasma TG (Figure 2B) and did not improve hepatic steatosis and visceral fat deposition (Figure 2C). Campari tomato was more protective than lycopene alone in DIO-zebrafish, consistent with previous reports that tomato powder administration is more efficacious than lycopene supplement against serum TG elevation and lipid peroxidation in oxidative stress model of rat [27]. Lipid peroxidation is highly related to nonalcoholic steatosis and steatohepatitis in human [28] and our model of DIO-zebrafish probably contains the same mechanism. Tomato contains vitamins A, C, and E and several carotenoids such as α-, β-, and γ-carotenes, luteins, phytoene, and phytofluene [29-31]. Many of these nutrients have antioxidant property and in combination with lycopene may contribute to protect against lipid peroxidation. On the other hand, Ali et al. [27] reported that when tomato was fed to rats only lycopene and β-carotene were detected their liver tissues.

**Table 1:** Carotenoids, sugar contents, ascorbic acid, and NO_3_^- ^determination in Campari and regular tomato

	Lycopene (mg/100 g)	β-carotene (mg/100 g)	Brix (%)	Ascorbic acid (mg/100 g)	NO_3_^- ^(mg/Kg)
Regular tomato	1.48 ± 0.28	0.48 ± 0.09	5.10 ± 0.61	17.28 ± 2.59	32.04 ± 18.45
Campari tomato	3.22 ± 0.85 *	1.06 ± 0.21 *	8.08 ± 0.15 *	20.90 ± 4.23	7.30 ± 2.93 **

* *P *< 0.05; ** *P *< 0.01

### Transcriptome analysis of liver of DIO-zebrafish with tomatoes

**Figure 3:**
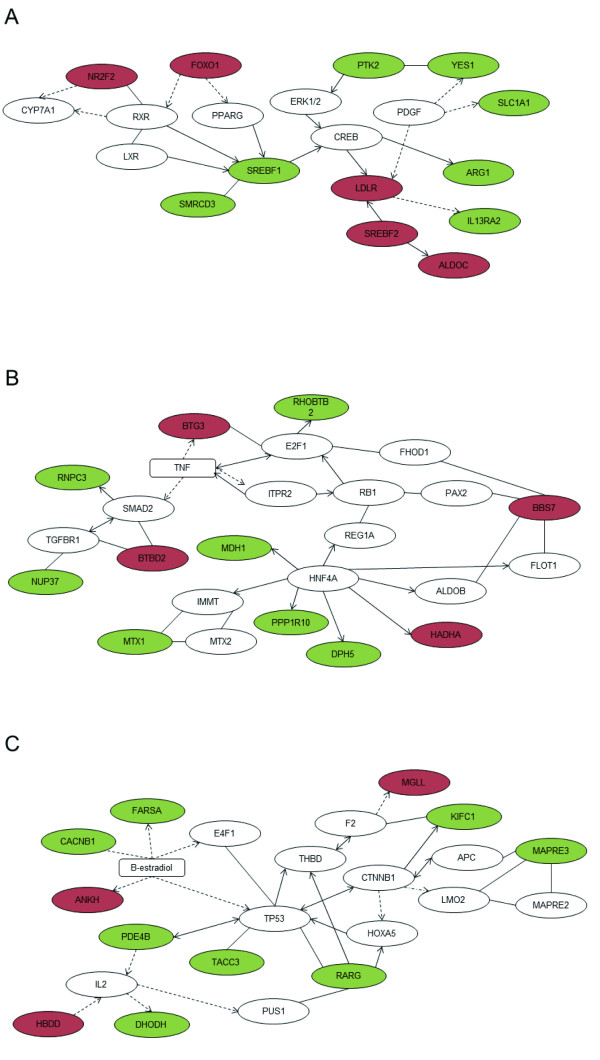
**Pathway analysis of liver from Campari tomato treatment**. Biological networks identified by IPA using 90 human orthologs altered in Campari tomato treatment. Intensity of node color indicates magnitude of upregulation (red) and downregulation (green). (A) Network 1, related to lipid metabolism. (B) Network 2, also related to lipid metabolism. (C) Network 3, related to cancer compromise. The scores of these networks are described in Table 2. Solid arrow, induction and/or activation; solid line without arrow head, binding; dashed arrow, suppression and/or inhibition.

To reveal the therapeutic mechanism of Campari tomato against hepatic steatosis, we conducted DNA microarray experiments using liver tissues from these zebrafish. In DIO-zebrafish fed Campari tomato expression of 116 and 52 probes was increased (> 1.3) and decreased (< 0.7), respectively, in comparison with regular tomato feeding (Additional file 6, Table S3). These 168 probes were converted to zebrafish genes by BLAST analysis [32], which revealed the genes corresponded to 90 human orthologs. For the validation of the results of DNA microarray experiments, quantitative RT-PCR was conducted (Table 2). Analysis of the genes with altered expression by Gene Ontology category using GOstat [33] revealed that 13.3% of their human orthologs (12 in 90 orthologs) are involved in lipid metabolism, including phospholipid binding (Table 3). Next, we applied these 90 gene altered by Campari tomato to construct functional networks by Ingenuity Pathway Analysis (IPA, Ingenuity Systems, CA, USA), and thereby identified three statistically significant networks each containing ≥10 altered genes (Table 4 and Figure 3A-C).

**Table 2:** QPCR of genes from biological networks in Campari tomato treatment compared with regular strain

gene	QPCR	Microarray data
aldoca	1.38 ± 0.46 *	2.23 **
foxo1	2.42 ± 0.59 **	n.d.
ldlr	1.53 ± 0.31 *	1.95 **
nr2f2	2.34 ± 0.53 **	2.85 **
pde4ba	0.13 ± 0.35 **	0.06 **
rarga	0.10 ± 0.22 **	0.06 **
srebf1	0.49 ± 0.18 *	n.d.
srebf2	1.31 ± 0.52 *	2.32 **

n = 4, * *P *< 0.05; ** *P *< 0.01

**Table 3:** Ontology analysis of genes with altered expression in the Campari tomato-fed fish relative to regular strain tomato-fed fish

Group	GO ID	GO Terms	Genes	*P*-Value
Lipid	GO:0006629	lipid metabolic process	ldlr stat5b cel hadha mgll nr2f2 srebf2	0.0757
	GO:0008289	lipid binding	gas1 pebp1 ncf1 hadha rassf1 rtn4rl1	0.0468
	GO:0008202	steroid metabolic process	ldlr stat5b cel srebf2	0.0468
	GO:0005543	phospholipid binding	gas1 pebp1 ncf1 rtn4rl1	0.0795
	GO:0016125	sterol metabplic process	ldlr cel srebf2	0.0468
	GO:0008203	choresterol metabolic process	ldlr cel srebf2	0.0407

Carbohydrate	GO:0008643	carbohydrate transport	yes1 slc2a8 slc2a12	0.0468

	GO:0015758	glucose transport	yes1 slc2a8	0.0468
Other	GO:0022892	substrate-specific transporter activity	hbd tomm7 ankh slc2a8 slc2a12 ldlr accn4 hbz cacnb1 slc1a1	0.0774
	GO:0031090	organelle membrane	mtx1 oxa1l dhodh nup37 tomm7 slc2a8 hadha srebf2 nupl2	0.0809
	GO:0007049	cell cycle	gas1 stat5b btg3 chfr kifc1 mapre3 rassf1	0.0566
	GO:0033036	macromolecule localization	mtx1 oxa1l vangl2 nup37 ppp1r10 tomm7 nupl2	0.0865

**Table 4:** Top networks from pathway analysis of Campari tomato vs. regular tomato

ID	Associated Network Functions	Score
1	Gene Expression, Lipid Metabolism, Small Molecule Biochemistry	47
2	Endocrine System Disorders, Metabolic Disease, Gene Expression	25
3	Cancer, Hematological System Development and Function, Cell Cycle	19

In the biological network of lipid metabolism (Figure 3A), sterol regulatory element-binding transcription factor 1 (*srebf1*) was downregulated. SREBF1 is the most important transcriptional factor regulating de novo lipogenesis in the liver, and plays a considerable role in the pathogenesis of nonalcoholic fatty liver disease [34,35]. *Srebf1 *is induced by heterodimerization of liver X receptor (LXR) and retinoid X receptor (RXR) [36]. RXR signalling mainly depends on retinoic acid and its precursor vitamin A and its precursor, β-carotene. However, since Campari tomato contains more β-carotene than regular tomato, downregulation of *srebf1 *seems contradictory. However, β-carotene inhibits atherogenesis and fatty liver formation in LDL receptor knockout mice [18] and reduces lipid storage capacity in adipocytes [17] through decrease of peroxisome proliferator-activated receptor (PPAR)-γ activity. To solve this paradoxical problem of *srebf1 *decrease by Campari tomato, we analyzed *forkhead box O1 *(*foxo1*) expression (Table 4; only detected in qPCR data). Resveratrol, a polyphenol present in peanuts and grapes, was reported to alleviate alcoholic fatty liver by inhibiting *Srebf1 *expression via Foxo1 signalling pathway [37]. This seems very similar to our result of Campari tomato. Foxo1 also represses PPAR-γ transactivation in adipocytes [38], which might imply that resveratrol-like phytoalexin, including resveratrol of tomato [39], exerted lipid-lowering property further to β-carotene and lycopene in Campari tomato. In contrast to *srebf1*, *srebf2 *was slightly increased. It is known that decreased intracellular cholesterol by statin leads to increased gene expression of *srebf2 *and active form of SREBF2 [40]. A similar mechanism was inferred for Campari tomato in DIO-zebrafish. Nuclear receptor subfamily 2, group F, member 2 (*nr2f2*) was also upregulated by Campari tomato. NR2F2 and RXR activate and bind the rat cholesterol 7 alpha-hydroxylase (CYP7A1) gene, a rate-limiting enzyme in the synthesis of bile acid from cholesterol via the classic pathway [41]. This also indicates activation of RXR signalling by β-carotene in Campari tomato.

The third network identified was related to cancer. It is well known that fatty liver caused by obesity may develop into nonalcoholic hepatitis then liver cancer, and dietary administration of lycopene and tomato extracts inhibit nonalcoholic hepatitis-promoted hepatocarcinogenesis in rat models induced by low volume of hepatic carcinogen diethylnitrosamine with high-fat diet [9]. The mechanism of inhibition of carcinogenesis is thought dependent on reduction of oxidative stress, mainly by NF-E2-related factor-2 and heme oxygenase-1. However, our observed Campari tomato effects on DIO-zebrafish seem related to retinoic acid receptor (RAR) signalling, especially RAR-γ in this network of cancer compromise. RAR-γ is a ligand-activated transcription factor for retinoic acid consisting of heterodimers of RARs and RXR. RAR-γ forms complexes with RAR-β that directly activate HOXA5 [42] and may consequently increase expression of TP53 [43], as seen in this network. Whereas high-fat diet induces fatty liver and *Rar-γ *expression in mouse liver [44], Campari tomato decreased rar-γ expression versus regular strain in DIO-zebrafish. RAR-γ has oncogenic potential in hepatocellular carcinoma according to cancer xenograft model [45]. RAR-β, contrariwise, is a tumor suppressor and transactivated by β-carotene and beta-apo-14'-carotenoic acid primarily via its conversion to retinoic acid [46]. Although the mechanism of suppression of *rar-γ *by Campari tomato requires further elucidation, the prospective effects of cancer prevention seem similar to RAR-β induction by β-carotene. Previous studies reported protective effects of tomatoes and their components against prostate, gastric, breast, and lung cancers [47-50]. Further study is required to investigate whether Campari tomato possesses higher anticancer properties in comparison with regular tomato.

## Conclusions

Our observations of transcriptome profiles demonstrate powerful lipid-lowering property of Campari tomato in DIO-zebrafish through downregulation of gene expression related to lipogenesis. DIO-zebrafish could discriminate different anti-obesity effects of vegetables and may be used to identify action mechanisms against obesity-related diseases, especially fatty liver disease. This is the first study that used zebrafish for food evaluation.

## List of Abbreviations

CVD: cardiovascular disease; mpf: months postfertilization; CYP7A1: cholesterol 7 alpha-hydroxylase; DIO-zebrafish: diet-induced obesity zebrafish; DPPH: 1, 1-Dipehnyl-2-picrylhydrazyl; FOXO1: forkhead box O1; HOXA5: homeobox A5; LDL: low-density lipoprotein; LXR: liver X receptor; NR2F2: nuclear receptor subfamily 2: group F: member 2; PPAR-γ: proliferator-activated receptor gamma; RAR-β: retinoic acid receptor beta; RAR-γ: retinoic acid receptor gamma; RXR: retinoid X receptor; SREBF1: sterol regulatory element-binding transcription factor 1; SREBF2: sterol regulatory element-binding transcription factor 2; TG: triacylglyceride; TP53: tumor protein p53.

## Competing interests

The authors declare that they have no competing interests.

## Authors' contributions

TTI and YS carried out the whole animal studies and drafted the manuscript. LZ and TO participated in the histological analysis. JK and YN carried out the DNA microarray experiments, participated in the bioinformatics analysis, and performed the statistical analyses. NN and TTN conceived the study, participated in its design and coordination, and helped to draft the manuscript. All authors read and approved the final manuscript.

## Authors' information

Toshiyuki Tainaka is a director in developmental section of Delica Foods, and a postgraduate student in Graduate School of Medicine, Mie University. The focus of Mr. Tainaka's research is the health-promoting function of vegetables.

Yasuhito Shimada, MD, is an assistance professor at the Department of Molecular and Cellular Pharmacology, Pharmacogenomics and Pharmacoinformatics, Graduate School of Medicine, Mie University. The focus of Dr. Shimada's research is studying for preventing obesity using a diet-induced obesity model of zebrafish.

Yuhei Nishimura, MD, PhD, is a lecturer in the Department of Molecular and Cellular Pharmacology, Pharmacogenomics and Pharmacoinformatics, Graduate School of Medicine, Mie University. Dr Nishimura's research interests are the bioinformatics and pharmacoinformatics.

Norihio Nishimura, PhD, is a professor in the Department of Translational Medical Science, Graduate School of Medicine, Mie University. Dr Nishimura's research interests are the agricultural biotechnology and food science.

Toshio Tanaka, MD, PhD, is a professor in the Department of Molecular and Cellular Pharmacology, Pharmacogenomics and Pharmacoinformatics, Graduate School of Medicine, Mie University. The focus of Dr Tanaka's research is to identify drug targets using pharmacogenomics approach. Dr. Tanaka has published over 100 peer-reviewed research articles and reviews in international journals.

## Supplementary Material

Additional file 1 **Figure S1**. Photographs of the vegetables used in this study. Click here for file

Additional file 2 **Table S1**. Primer sequences for QPCR. Click here for file

Additional file 3 **Table S2. Body weight and plasma TG of DIO-zebrafish treated with vegetables for 2 weeks**. At 3.5 months postfertilization (mpf) zebrafish overfed or normally fed with *Artemia *were orally fed vegetable-containing flake food (2 mg/day) 3 times daily (6 mg/day) for 2 weeks. *P < 0.05; **P < 0.01 vs. vehicle administered with overfeeding; ‡*P *< 0.05 vs. normal feeding. Click here for file

Additional file 4 **Figure S2**. Fasting blood glucose of normally fed and overfed zebrafish with and without tomato supplementation. Click here for file

Additional file 5 **Figure S3**. The DPPH radical scavenging activity of tomato-containing fish foods. Click here for file

Additional file 6 **Table S3**. 168 probes were altered by Campari tomato in comparison to regular tomato. Click here for file
