# Supplementary material for: Transcriptome analysis of anti-fatty liver action by Campari tomato using a zebrafish diet-induced obesity model
Source: Nutr Metab (Lond). 2011 Dec 13;8:88. doi: 10.1186/1743-7075-8-88 (PMC3275548; doi:10.1186/1743-7075-8-88)
Supplement: Additional file 1 — Figure S1. Photographs of the vegetables used in this study. [file 1743-7075-8-88-S1.PPT]

## Slide 1
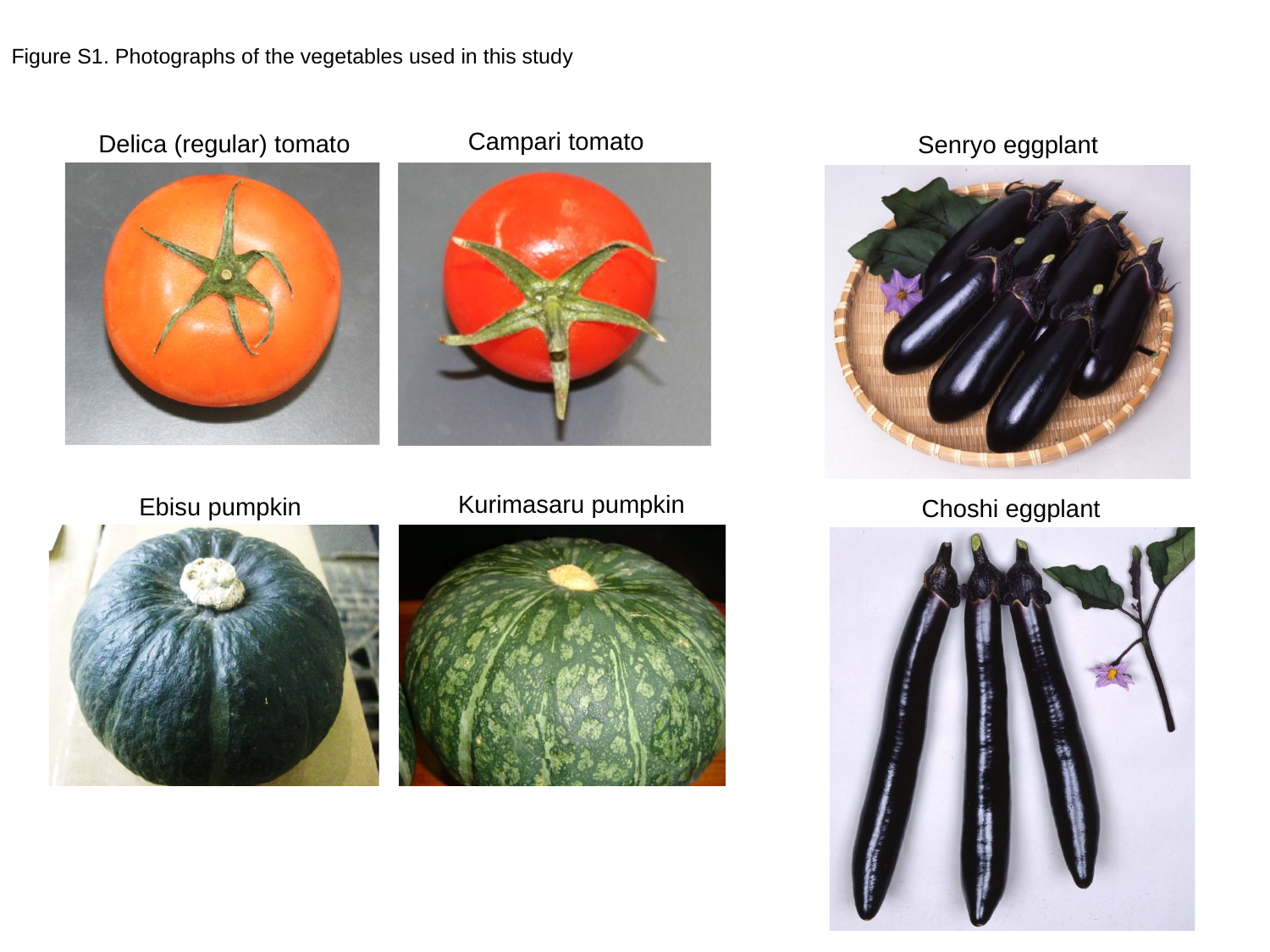

Figure S1. Photographs of the vegetables used in this study
Campari tomato
Delica (regular) tomato
Senryo eggplant
Kurimasaru pumpkin
Ebisu pumpkin
Choshi eggplant
