# Supplementary material for: Transcriptome analysis of anti-fatty liver action by Campari tomato using a zebrafish diet-induced obesity model
Source: Nutr Metab (Lond). 2011 Dec 13;8:88. doi: 10.1186/1743-7075-8-88 (PMC3275548; doi:10.1186/1743-7075-8-88)
Supplement: Additional file 2 — Table S1. Primer sequences for QPCR. [file 1743-7075-8-88-S2.PPT]

## Slide 1
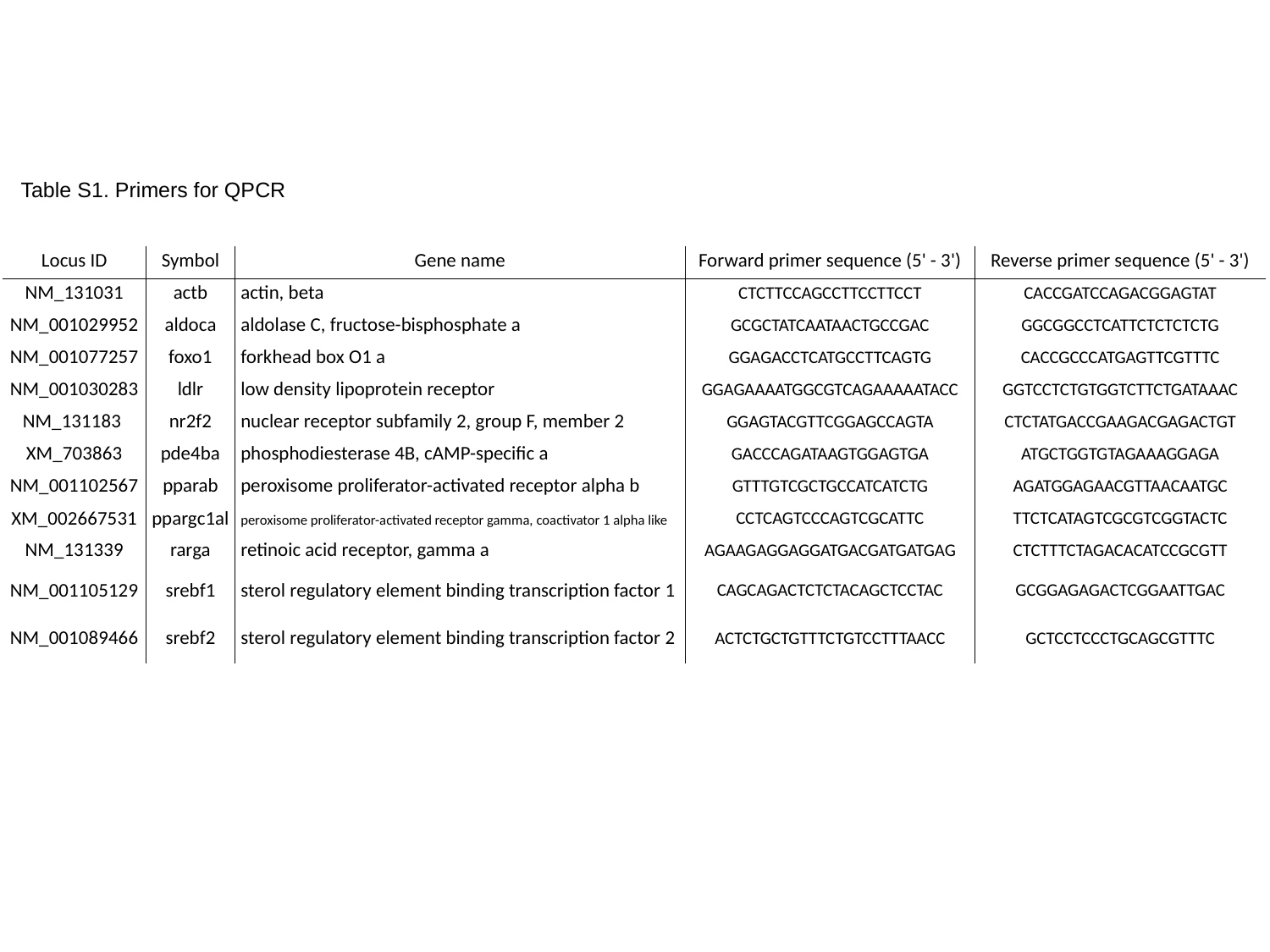

Table S1. Primers for QPCR
| Locus ID | Symbol | Gene name | Forward primer sequence (5' - 3') | Reverse primer sequence (5' - 3') |
| --- | --- | --- | --- | --- |
| NM\_131031 | actb | actin, beta | CTCTTCCAGCCTTCCTTCCT | CACCGATCCAGACGGAGTAT |
| NM\_001029952 | aldoca | aldolase C, fructose-bisphosphate a | GCGCTATCAATAACTGCCGAC | GGCGGCCTCATTCTCTCTCTG |
| NM\_001077257 | foxo1 | forkhead box O1 a | GGAGACCTCATGCCTTCAGTG | CACCGCCCATGAGTTCGTTTC |
| NM\_001030283 | ldlr | low density lipoprotein receptor | GGAGAAAATGGCGTCAGAAAAATACC | GGTCCTCTGTGGTCTTCTGATAAAC |
| NM\_131183 | nr2f2 | nuclear receptor subfamily 2, group F, member 2 | GGAGTACGTTCGGAGCCAGTA | CTCTATGACCGAAGACGAGACTGT |
| XM\_703863 | pde4ba | phosphodiesterase 4B, cAMP-specific a | GACCCAGATAAGTGGAGTGA | ATGCTGGTGTAGAAAGGAGA |
| NM\_001102567 | pparab | peroxisome proliferator-activated receptor alpha b | GTTTGTCGCTGCCATCATCTG | AGATGGAGAACGTTAACAATGC |
| XM\_002667531 | ppargc1al | peroxisome proliferator-activated receptor gamma, coactivator 1 alpha like | CCTCAGTCCCAGTCGCATTC | TTCTCATAGTCGCGTCGGTACTC |
| NM\_131339 | rarga | retinoic acid receptor, gamma a | AGAAGAGGAGGATGACGATGATGAG | CTCTTTCTAGACACATCCGCGTT |
| NM\_001105129 | srebf1 | sterol regulatory element binding transcription factor 1 | CAGCAGACTCTCTACAGCTCCTAC | GCGGAGAGACTCGGAATTGAC |
| NM\_001089466 | srebf2 | sterol regulatory element binding transcription factor 2 | ACTCTGCTGTTTCTGTCCTTTAACC | GCTCCTCCCTGCAGCGTTTC |
