# Supplementary figures and images for: Transcriptome analysis of anti-fatty liver action by Campari tomato using a zebrafish diet-induced obesity model
Source: Nutr Metab (Lond). 2011 Dec 13;8:88. doi: 10.1186/1743-7075-8-88 (PMC3275548; doi:10.1186/1743-7075-8-88)

## Slide 1
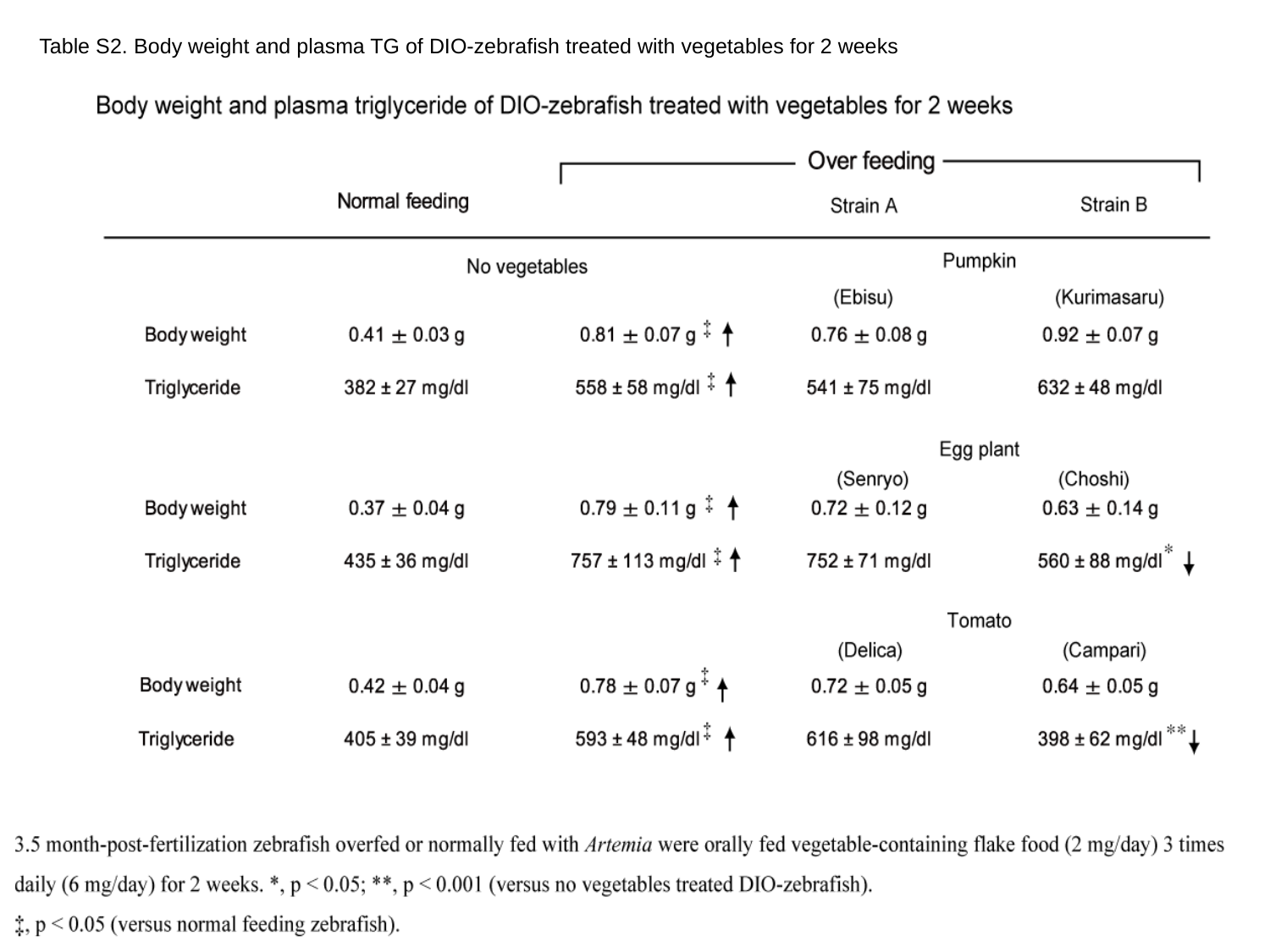

Table S2. Body weight and plasma TG of DIO-zebrafish treated with vegetables for 2 weeks

Supplement: Additional file 3 — Table S2. Body weight and plasma TG of DIO-zebrafish treated with vegetables for 2 weeks. At 3.5 months postfertilization (mpf) zebrafish overfed or normally fed with Artemia were orally fed vegetable-containing flake food (2 mg/day) 3 times daily (6 mg/day) for 2 weeks. *P < 0.05; **P < 0.01 vs. vehicle administered with overfeeding; ‡P < 0.05 vs. normal feeding. [file 1743-7075-8-88-S3.PPT]
