# Supplementary material for: Transcriptome analysis of anti-fatty liver action by Campari tomato using a zebrafish diet-induced obesity model
Source: Nutr Metab (Lond). 2011 Dec 13;8:88. doi: 10.1186/1743-7075-8-88 (PMC3275548; doi:10.1186/1743-7075-8-88)
Supplement: Additional file 4 — Figure S2. Fasting blood glucose of normally fed and overfed zebrafish with and without tomato supplementation. [file 1743-7075-8-88-S4.PPT]

## Slide 1
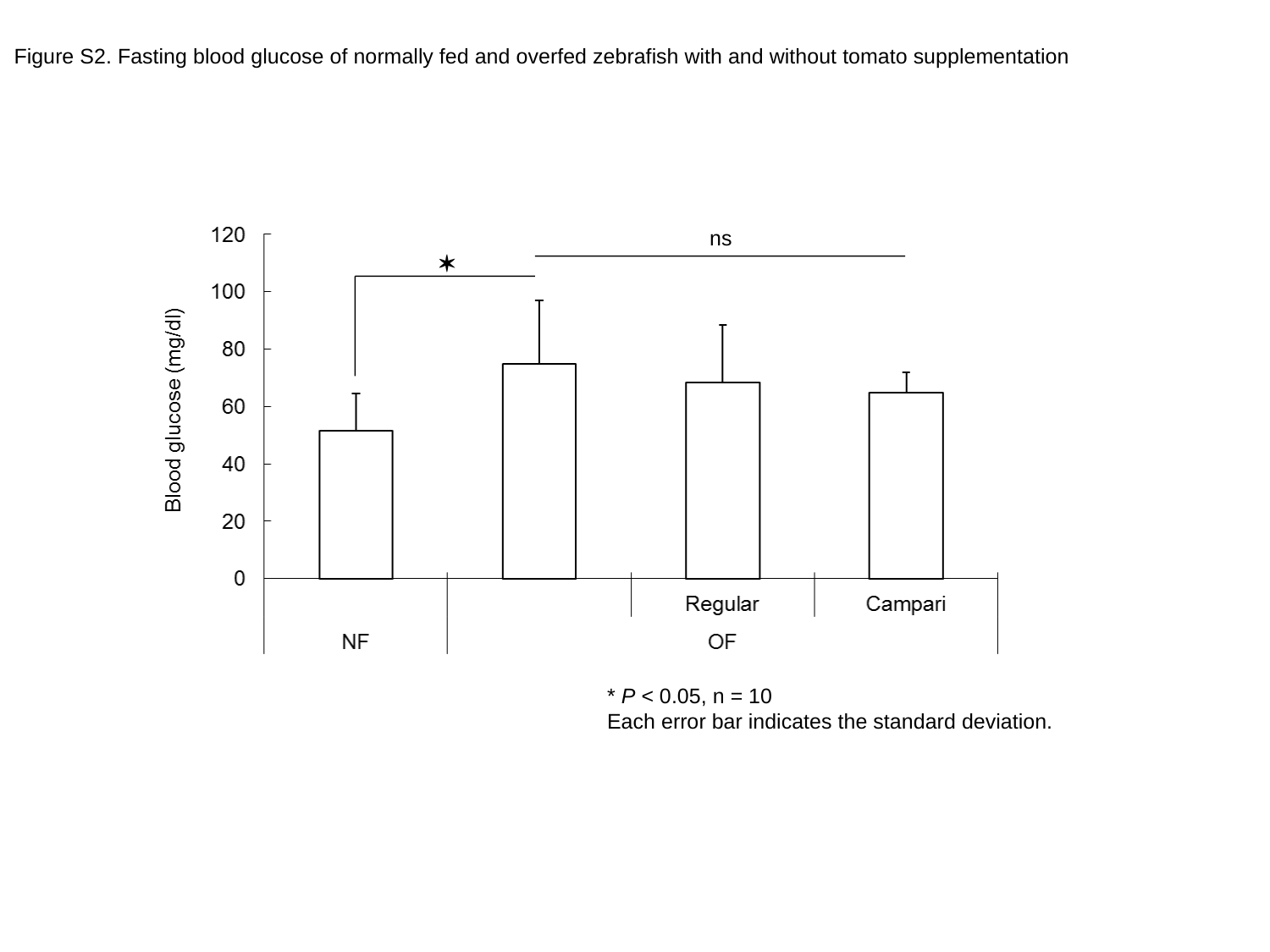

Figure S2. Fasting blood glucose of normally fed and overfed zebrafish with and without tomato supplementation
ns

* P < 0.05, n = 10
Each error bar indicates the standard deviation.
