# Supplementary material for: Transcriptome analysis of anti-fatty liver action by Campari tomato using a zebrafish diet-induced obesity model
Source: Nutr Metab (Lond). 2011 Dec 13;8:88. doi: 10.1186/1743-7075-8-88 (PMC3275548; doi:10.1186/1743-7075-8-88)
Supplement: Additional file 5 — Figure S3. The DPPH radical scavenging activity of tomato-containing fish foods. [file 1743-7075-8-88-S5.PPT]

## Slide 1
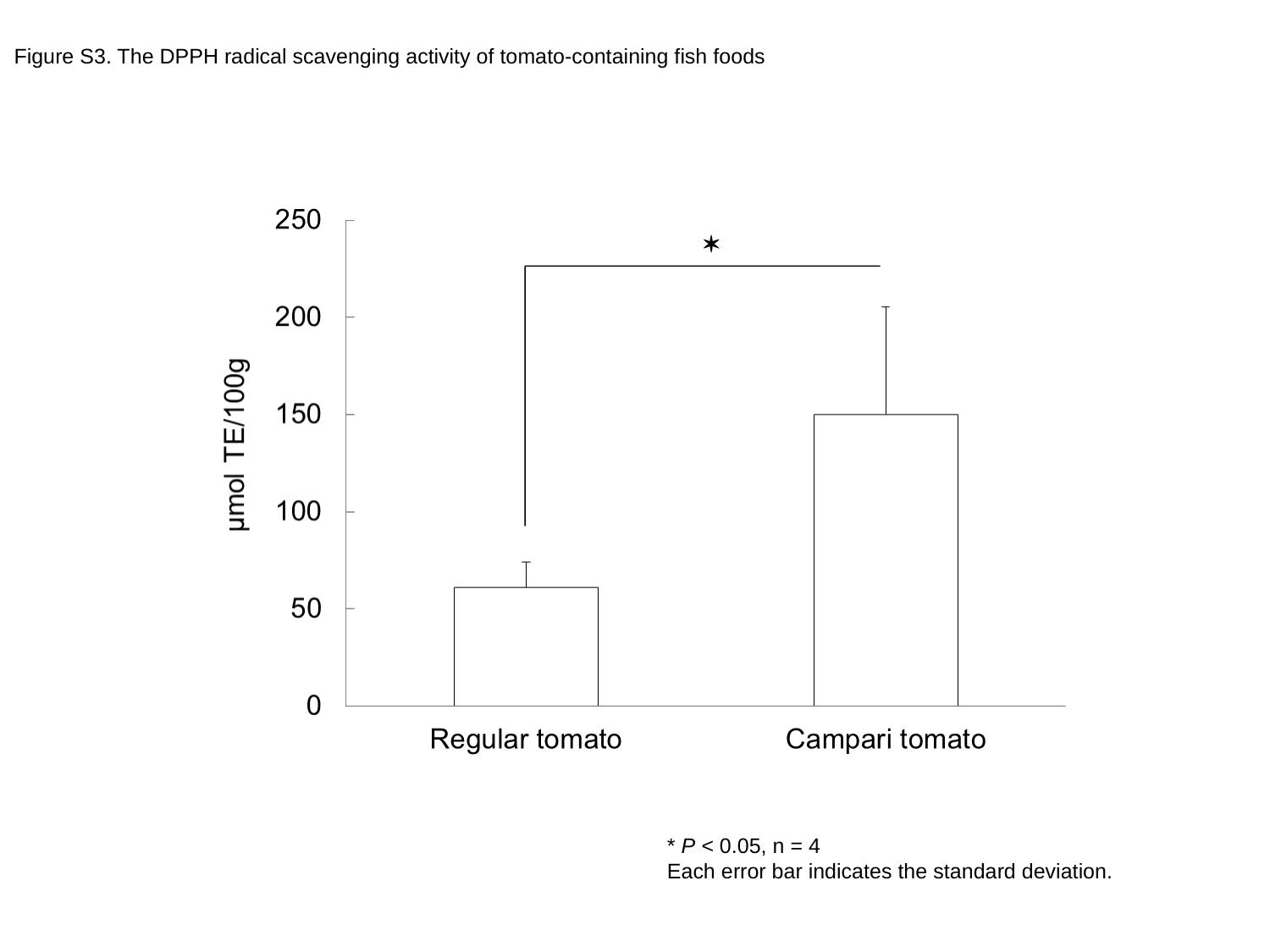

Figure S3. The DPPH radical scavenging activity of tomato-containing fish foods

* P < 0.05, n = 4
Each error bar indicates the standard deviation.
